# Combination GITR targeting/PD-1 blockade with vaccination drives robust antigen-specific antitumor immunity

**DOI:** 10.18632/oncotarget.16605

**Published:** 2017-03-27

**Authors:** Daniel O. Villarreal, Diana Chin, Melissa A. Smith, Leopoldo L. Luistro, Linda A. Snyder

**Affiliations:** ^1^ Oncology Discovery, Janssen Research and Development, Spring House, PA 19477, USA

**Keywords:** vaccines, GITR, PD-1, immunotherapy, immuno-oncology

## Abstract

Tumor progression is facilitated immunologically by mechanisms that include low antigen expression, an absence of coimmunostimulatory signals, and the presence of regulatory T cells (Tregs), all of which act to suppress and restrict effector T cells in the tumor. It may be possible to overcome these conditions by a combination of modulatory immunotherapy agents and tumor-antigen targeting to activate and drive effective antitumor T cell responses. Here, we demonstrated that co-administration of aGITR and aPD-1 monoclonal antibodies (mAb) in combination with a peptide vaccine (Vax) in mice bearing established tumors significantly delayed tumor growth and induced complete regression in 50% of the mice. This response was associated with increased expansion and functionality of potent Ag-specific polyfunctional CD8^+^ T cells, reduced Tregs, and the generation of memory T cells. Tumor regression correlated with the expansion of tumor-infiltrating antigen-specific CD8^+^ effector memory T cells, as depletion of this cell population significantly reduced the effectiveness of the triple combination Vax/aGITR/aPD-1 therapy. These findings support the concept that dual aGITR/aPD-1 combination with cancer vaccines may be a novel strategy against poorly immunogenic tumors.

## INTRODUCTION

The generation of potent, cytolytic CD8^+^ T lymphocyte (CTL) responses is critical for developing an effective antitumor response. To counteract these cells, tumors use multiple inhibitory mechanisms to suppress effector immune responses, leading to the attenuation and exhaustion of cytolytic tumor-infiltrating lymphocytes (TILs) [1–5]. For example, programmed death 1 (PD-1) is a key immune checkpoint receptor expressed on the surface of activated T cells that, when engaged with its ligands in the tumor microenvironment, downregulates anti-tumor T cell activity [6–8]. Recent clinical trials have demonstrated that monoclonal antibodies (mAbs) that block PD-1 reinvigorate TILs and are providing clinical benefit to patients with melanoma or lung cancer, among other malignancies [9–10]. These results have stimulated enormous interest in cancer immunotherapy, but it remains true that when such mAbs are administered as monotherapies, only a subset of patients achieves clinical benefit [9–10]. Therefore, the development of more effective approaches or combinatorial strategies is required to target the many mechanisms of tumor-induced T cell immunosuppression.

The generation of effective antitumor responses will not only require a blockade of co-inhibitory pathways, but novel modalities to increase the number of immune effector cells. One such approach is to target costimulatory molecules, such as the TNF receptor family member GITR. An agonist GITR antibody has demonstrated success in enhancing antitumor immunity in several preclinical models [11–16]. Mechanistic studies have revealed that co-stimulatory effects of GITR-triggering on T cells, both conventional CD4^+^ and CD8^+^ T cells, increases their proliferation, activation, and boosts their cytokine production [12–13]. GITR ligation has also been demonstrated to inhibit the expansion and suppressive activity of CD4 regulatory T cells (Tregs) [11–13, 17]. A recent study has demonstrated that anti-GITR (aGITR) and anti-PD-1 (aPD-1) can synergize as a combination therapy to augment antitumor activity [18]. Although the combined treatment induced antitumor immunity, the therapy led to minimal tumor clearance, possibly due to its limited ability to overcome T cell tolerance and drive potent tumor-specific CD8^+^ T cell responses in well-established immunosuppressive tumor microenvironments (TME) [18–20]. Thus, the priming and expansion of tumor-reactive CD8^+^ T cells will be paramount to overcome T-cell anergy. One approach to overcome this limitation would require the administration of these therapies with an Ag-specific vaccine. The ability of combination aGITR/aPD-1 to influence the Ag-specific CD8^+^ T cell immune responses and work in synergy with vaccines remains to be explored. Therefore, we investigated whether combination therapy that brings together three stings of power, PD1 blockade and GITR targeting with a vaccine would enhance tumor-specific CD8^+^ T immunity in a stringent, palpable B16 treatment model.

The well-studied B16 melanoma cell lines are poorly immunogenic and possess many characteristics of analogous tumors found in patients [21–25], and therefore, are considered to be good models to develop combination strategies against poorly immunogenic tumors. We hypothesized and confirmed that using a peptide vaccine (Vax) targeting OVA as a model tumor antigen in combination with aGITR/aPD-1 mAb therapy would induce the expansion of cytolytic antigen (Ag)-specific tumor-reactive CD8^+^ T cells, reduce regulatory T cells, and thus prolong survival in mice with established, palpable B16-OVA tumors. As such, these findings show that the clinical combination of multiple therapeutic strategies that exploit different tumor immune vulnerabilities may offer a novel strategy to improve tumor immunotherapy in patients with cancer.

## RESULTS

### Combined aGITR and aPD1 therapy with vaccination induced robust antigen-specific CD8^+^ T cell expansion, function, and differentiation in non-tumor bearing mice

**Figure 1:**
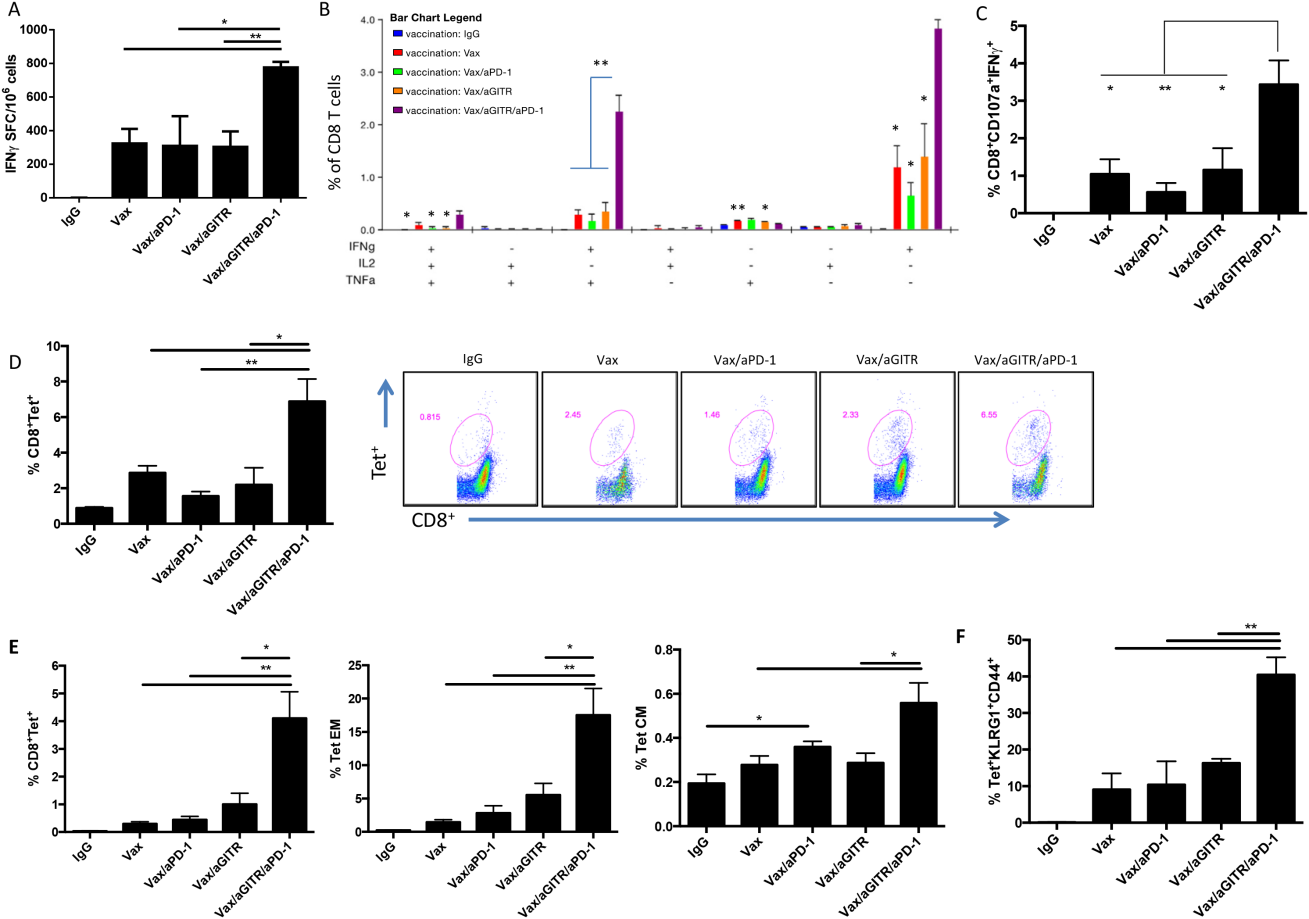
Combination aGITR/aPD-1 therapy with vaccination boosts the expansion, function and differentiation of Ag-specific CD8^+^ T cells Naïve B6 non-tumor bearing mice (n = 5/group) were immunized once with Vax (day 0), along with mono- or combination therapy: 200 μg aGITR or control rat IgG on days 0, 3 and 6, and 200 μg of aPD-1 on days 3, 6, 9 and 12. Desired immune responses were monitored at day 7 (d7) and day 14 (d14) in the blood and/or spleen. **(A)** ELISpot analysis of IFNγ-secreting T cells from spleens of mice stimulated with OVA_257-264_-specific peptide (d7). **(B)** column graphs show polyfunctional subpopulations of single-, double- and triple-positive CD8^+^ T cells releasing effector cytokines IFNγ, TNFα, and IL-2 to OVA_257-264_ stimulation in the spleen (d7). **(C)** profile of the cytolytic phenotype (d7). **(D)** OVA-specific CD8^+^ T cells in peripheral blood (d7). Dot plots are representative of each group shown in **(D)**. **(E)** OVA-specific CD8^+^ T cells in peripheral blood at d14. **(E-F)**, differentiation of OVA tetramer-specific CD8^+^ memory T cells in the blood from treated mice at d14 after immunization. Tet^+^ were derived from EM: effector memory (CD8^+^CD44^+^CD62L^−^); CM: central memory (CD8^+^CD44^+^CD62L^+^). KLRG1^+^ cell are derived from CD8^+^CD44^+^Tet^+^. Each of the above experiments was repeated at least two times with similar results. *P<0.05; **P<0.01; ***P<0.001. Error bars indicate SEM.

We first assessed the mechanisms by which combination therapy targeting GITR with PD-1 blockade augments Ag-specific CD8^+^ T cell responses in a vaccine setting. To address this, non-tumor bearing mice were immunized once with the OVA immunodominant CTL epitope OVA267-264 peptide vaccine (hereafter referred to as Vax) and treated with 200 μg aGITR on days 0, 3, and 6 and 200 μg aPD-1 on days 3, 6, 9, and 12. Combination Vax/aGITR/aPD-1 therapy augmented CD8^+^ effector function over controls, as evidenced by increased levels of splenic Ag-specific IFNγ ELISpot responses, polyfunctional CD8^+^ T cell responses, and increased levels of CD107a/IFNγ CD8^+^ T cells demonstrating cytolytic activity (Figures 1A, 1B, and 1C, respectively). Interestingly, the triple therapy elicited significantly higher frequencies of polyfunctional effector CD8^+^ T cells expressing single IFNγ, dual IFNγ/TNFα, and triple IFNγ/TNFα/IL-2, as compared with the other treatments and control groups (Figure 1B). By direct staining with OVA257-264 H-2K^b^-SIINFEKL tetramer, Vax/aGITR/aPD-1 amplified significantly the frequency of OVA tetramer-specific CD8^+^ T cell responses in the peripheral blood at day 7 and 14 (Figure 1D and 1E), suggesting the trafficking of target-specific CD8^+^ T cells. The high frequencies of effector cells secreting Th1 inflammatory cytokines are indicative that *in vivo* combination of aGITR/aPD-1 can enhance vaccine-induced Ag-specific CD8^+^ T cell responses.

We next determined the extent to which combination therapy skewed Ag-specific CD8^+^ T cell differentiation toward an effector versus memory phenotype, by surface expression of CD44 and CD62L, 14 days after vaccine priming. The phenotypic profile for central memory (CM) is typically CD44^+^ and CD62L^+^, and effector memory (EM) cells are CD44^+^ and CD62L^−^. We observed a significant increase in the tetramer OVA-specific EM and CM CD8^+^ T cell populations in mice given triple combination therapy, compared to other groups (Figure 1E). Furthermore, it has been highlighted that a predominant population KLRG1^+^CD8^+^ T cells are an optimal effector subset for protective immunity [26–28], and likely a vital subset that correlates with the efficacy of cancer immunotherapies [29–31]. Therefore, we characterized the phenotype of the Ag-specific CD8^+^ T cell population to express the cell surface expression of KLRG1 as a correlate. As shown in Figure 1F, the percentages of tetramer-specific KLRG1^+^ effector memory CD8^+^ T cells were significantly higher in the triple combination group compared with control groups. Together, these results demonstrate that aGITR/aPD-1 combination with vaccination can enhance the expansion and function of potent Ag-specific memory CD8^+^ T cells *in vivo*.

### Combination therapy with vaccination induced tumor regression and enhanced survival in tumor-bearing mice

**Figure 2:**
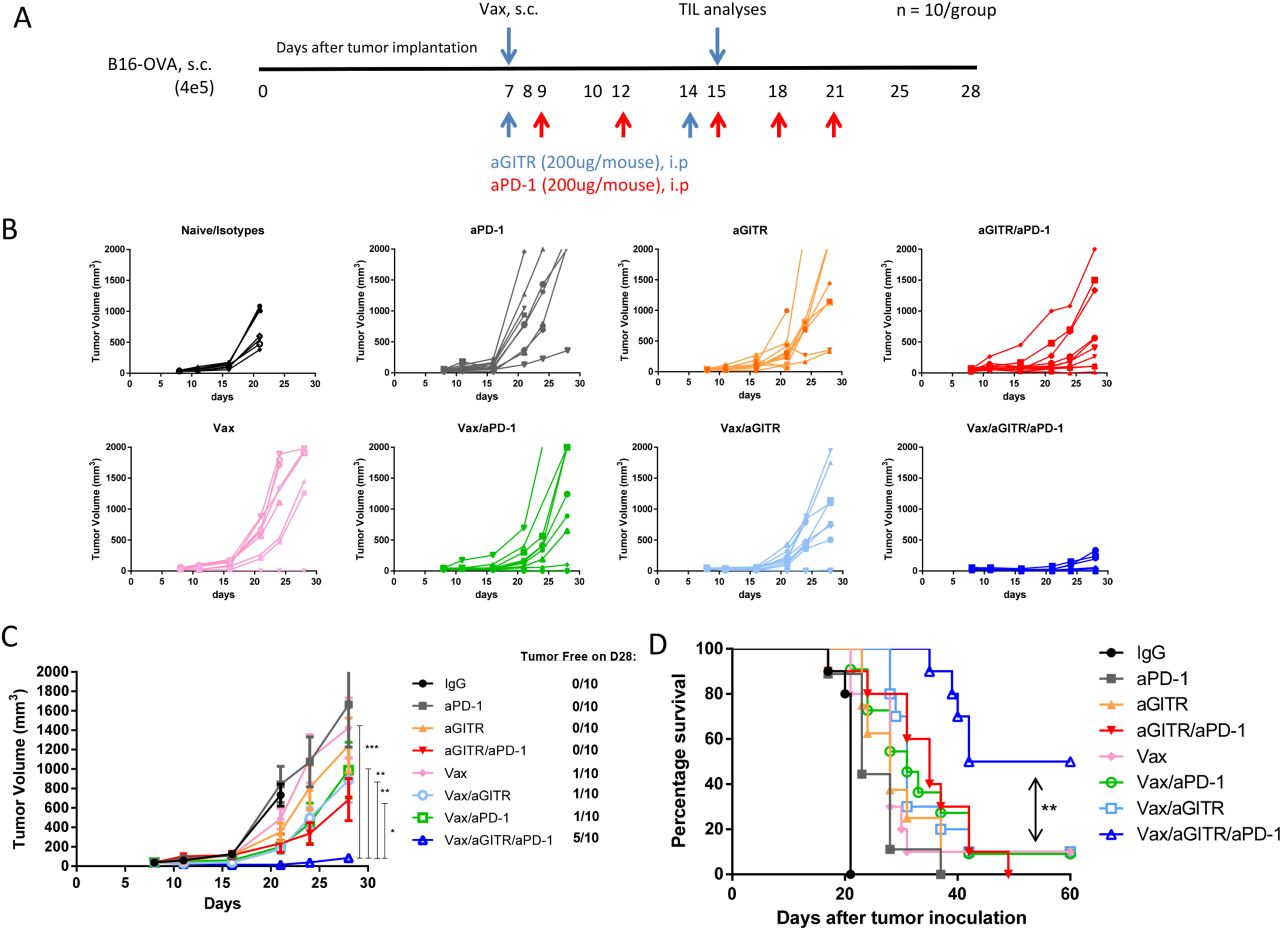
Combination aGITR/aPD-1 therapy with vaccination promotes B16-OVA tumor rejection in mice **(A)** B16-OVA established tumors (~30-40 mm^3^) were treated with the indicated treatments. **(B)** Individual tumor responses, group tumor measurements (mean +/− SEM, **(C)**) and survival **(D)** were monitored over time. Graph represents mean tumor volume per group of animals studied and chart indicates number of tumor-free/total **(C)**. Isotype-treated mice did not survive past day 21 due to severe morbidity. Graphs are representative results of 1 of 3 independent experiments. *P<0.05; **P<0.01; ***P<0.001.

Given the increase of Ag-specific effector CD8^+^ T cell responses induced by the triple combination therapy in the non-tumor bearing setting, we next asked whether the combination could induce an antitumor response using the poorly immunogenic B16-OVA melanoma model [21–25]. B16-OVA tumor cells were implanted into cohorts of naïve recipient B6 mice (n = 10/group). Seven days after implantation when tumors reached an average size of ~30-40 mm^3^, mice were randomized, and treated with the therapies as outlined in Figure 2A. There was no difference between Poly(I:C)/CpG alone treated group compared to IgG controlled group (Supplementary Figure 1). The antibody regimens without a vaccine slowed tumors modestly, but did not lead to tumor clearance, likely due to weak induction of Ag-specific T cells. Similarly, neither Vax alone or in combination with aGITR or aPD-1 mAbs resulted in greater than 10-20% survival. However, tumors in mice treated with Vax/aGITR/aPD-1 grew significantly slower than all other groups (Figure 2B-2C). Interestingly, the combination Vax/aGITR/aPD-1 therapy significantly enhanced tumor regression and survival in approximately 50% of mice over other combination therapies or vaccine alone (Figures 2C-2D). Taken together, the data shows that aGITR targeting and aPD-1 blockade combination can synergize with a vaccine to enhance overall survival.

### Combined Vax/aGITR/aPD-1 immunotherapy induces Ag-specific polyfunctional CD8^+^ T cells and reduces Treg population in tumors

**Figure 3:**
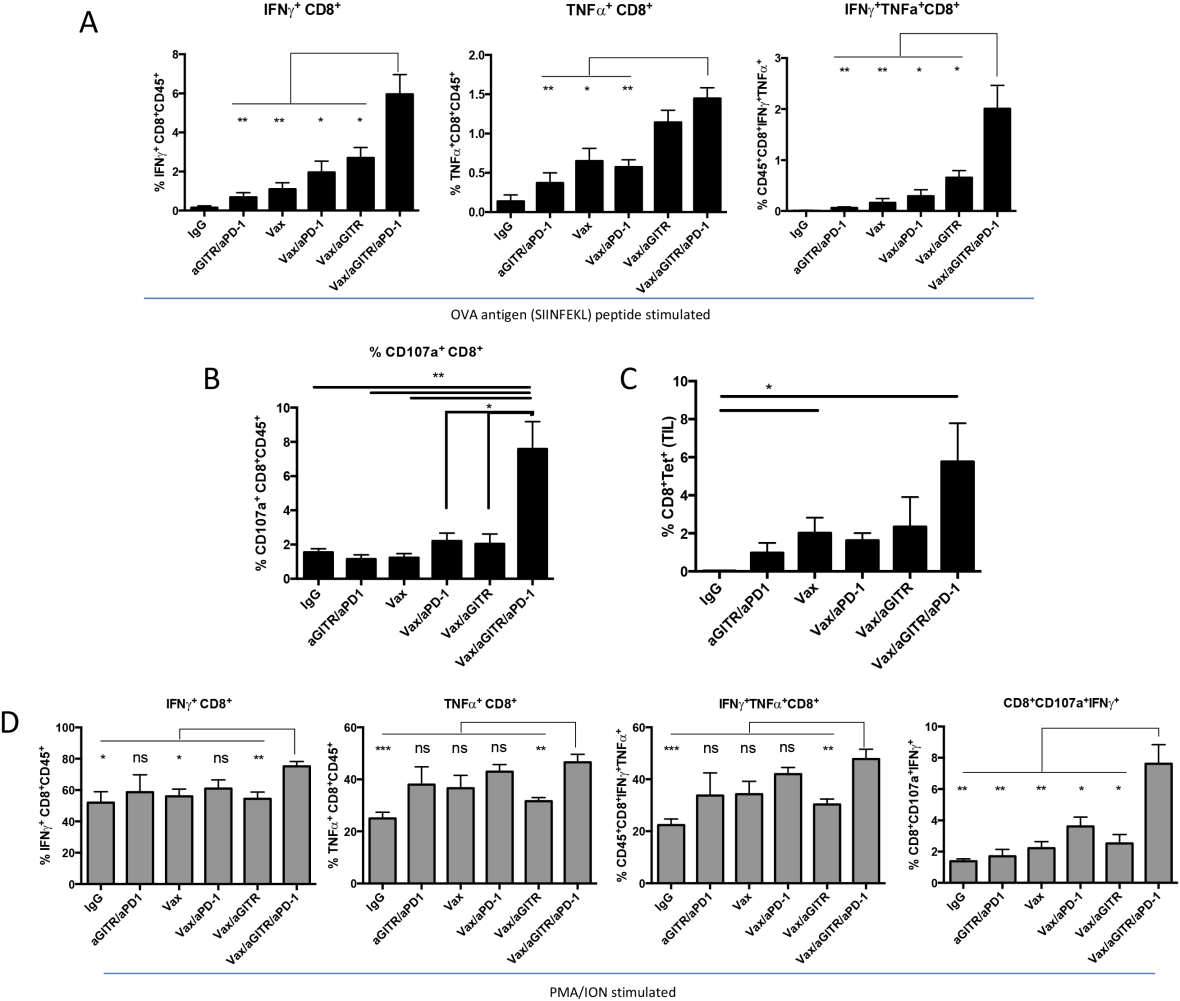
Combination Vax/aGITR/aPD-1 therapy synergized to enhance the frequency and function of vaccine-induced antigen-specific responses of CD8^+^ TILs Shown are summary data of the intracellular cytokine staining for IFNγ, TNFα, IFNγ/TNFα and CD107a/IFNγ in CD8^+^ TILS following OVA_257-264_ peptide stimulation **(A-B)** or with PMA/ION stimulation **(D)** 12 to 15 days after tumor implantation. **(C)** Bar graph shows the percentages of H2-K^b^-SIINFEKL-restricted OVA tetramer-specific CD8^+^ TILs of total CD45^+^ cells in the tumor. Experiments were repeated at least two times with similar results. All cell counts are relative and not absolute. *P<0.05; **P<0.01; ***P<0.001. Error bars indicate SEM of n = 4-5/group.

To understand the mechanism of action of the combination therapy, we next characterized the Ag-specific phenotype and functional response of CD8^+^ effector and CD4^+^ Tregs isolated from tumors following the various immunotherapies. Given the importance of multifunctional effector CD8^+^ T cell immunity in anti-tumor immunity [30–32], we measured the Ag-specific CD8^+^ T cell population and its expression of IFNγ and TNFα, in response to *ex vivo* OVA257-264 SIINFEKL peptide stimulation, 15 days after tumor implantation (Figure 3A). The Vax/aGITR/aPD-1 combination therapy significantly increased IFNγ and TNFα production from effector CD8^+^ T cells in tumors compared to all other groups (Figure 3A). Moreover, the Vax/aGITR/aPD-1 therapy showed a synergistic effect, as illustrated by the higher frequency of OVA-specific IFNγ/TNFα dual-positive CD8^+^ T cells within the tumor (Figure 3A). Given that cytolytic CD8^+^ CTLs are critical components in protection against tumors [30–32], we characterized the cytolytic potential of the cells to undergo degranulation, determined by the expression marker CD107a. We found that CD8^+^ tumor infiltrating lymphocytes (TILs) isolated from tumor-bearing mice treated with Vax/aGITR/aPD-1 had a significantly higher frequency of CD8^+^ T cells specific for OVA257-264 and expressing CD107a compared to controls, suggesting these T cells have greater potential to target tumor cells (Figure 3B). The triple combination also induced higher frequency of tetramer OVA-specific CD8^+^ T cells trafficking into the tumors (Figure 3C). Furthermore, a similar trend was seen with the frequency of CD8^+^ T cells secreting IFNγ, TNFα and/or expressing CD107a when stimulated with PMA/ION, indicating that the combination Vax/aGITR/aPD-1 induced more functional CD8^+^ T cell responses overall (Figure 3D). Interestingly, the Vax/aGITR/aPD-1 treated TILs stimulated with PMA/ION had higher frequencies of cytolytic CD8^+^ T cells coexpressing CD107a^+^IFNγ^+^. This correlates the substantial increase in cytolytic activity with significant control and/or regression of established tumors in the mice.

**Figure 4:**
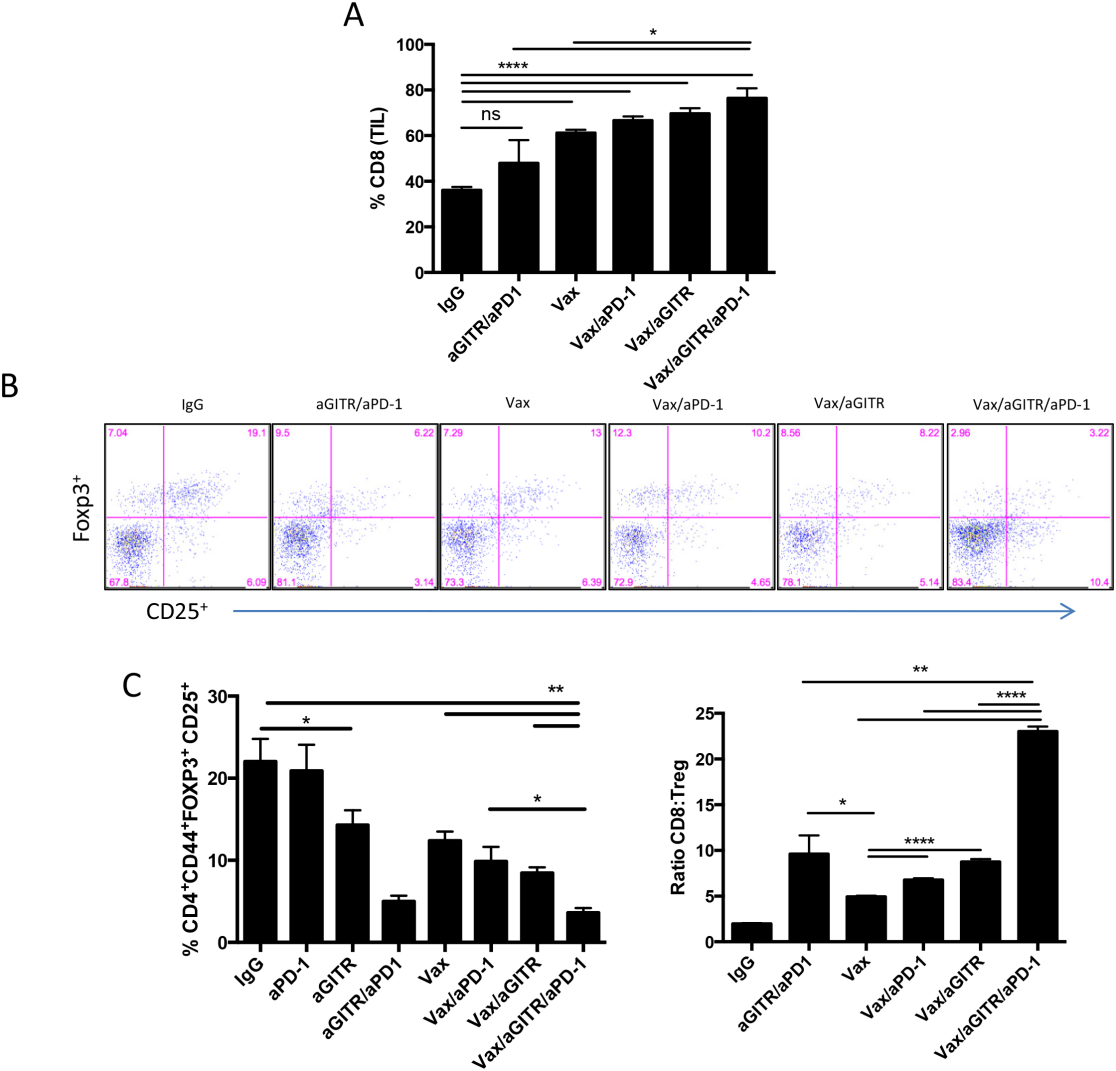
Combination Vax/aGITR/aPD-1 therapy enhances CD8^+^ T cell infiltration and reduces frequency of Tregs in B16-OVA tumors **(A-C)** cohorts of B16-OVA tumor-bearing mice were treated with Vax, aGITR, and/or PD-1 combinations (as in Figure 2). **(A)** CD8^+^ TILs as percentage of total CD45^+^ cells 15 days after tumor implantation. **(B-C)** Representative flow dot plots and summary data show the percentage of Tregs of CD45^+^ TILs and the ratio of CD8^+^ effector T cells to Tregs in the tumors of treated mice 15 days after tumor implantation. Statistical analyses are compared with Vax/aGITR/aPD-1. **(C)** CD8^+^ TILs as percentage of total CD45^+^ cells 15 days after tumor implantation. Results are representative of 2 to 3 independent experiments with 4 to 5 mice per group. All cell counts are relative and not absolute. *P<0.05; **P<0.01; ***P<0.001. Error bars indicate SEM.

We next sought to evaluate the effects of the combined Vax/aGITR/aPD-1 immunotherapy to reduce CD4^+^ Tregs in the tumors. When we monitored the Treg population at day 15 post-tumor implantation, both aGITR/aPD-1 and VAX/aGITR/aPD-1 immunotherapies similarly and significantly reduced the percentages of infiltrating Tregs in the tumors (Figure 4B-4C). These results indicate that combination with aGITR in both settings help facilitate better reduction of tumor infiltrating Tregs [11–16]. The triple combination overall showed better reduction of Tregs in the tumors compared to all treated groups. All immunotherapies, except aGITR/aPD1, strongly increased CD8^+^ T cell infiltration into the tumors (Figure 4A), likely due to the induction of Ag-specific T cell responses induced by the peptide vaccine as demonstrated in Figure 1 and Figure 3A. As a result, the CD8/Treg ratios within the tumor increased markedly, with the triple combination therapy being statistically superior to any other Ab combination therapy (Figure 4C), a response which has been described as a correlate for therapeutic efficacy in the melanoma model [33]. Collectively, the synergistic effects of the combination Vax/aGITR/aPD-1 to enhance tumor-reactive CTL responses, reduce Tregs, and drive higher ratios of effector T cells to Tregs in the tumors, may represent a more Ag-specific inflammatory microenvironment that is capable of mediating tumor clearance.

### Combination Vax/aGITR/aPD-1 therapy induced B16-OVA tumor rejection mediated by CD8^+^ T cells and elicited long-term memory

**Figure 5:**
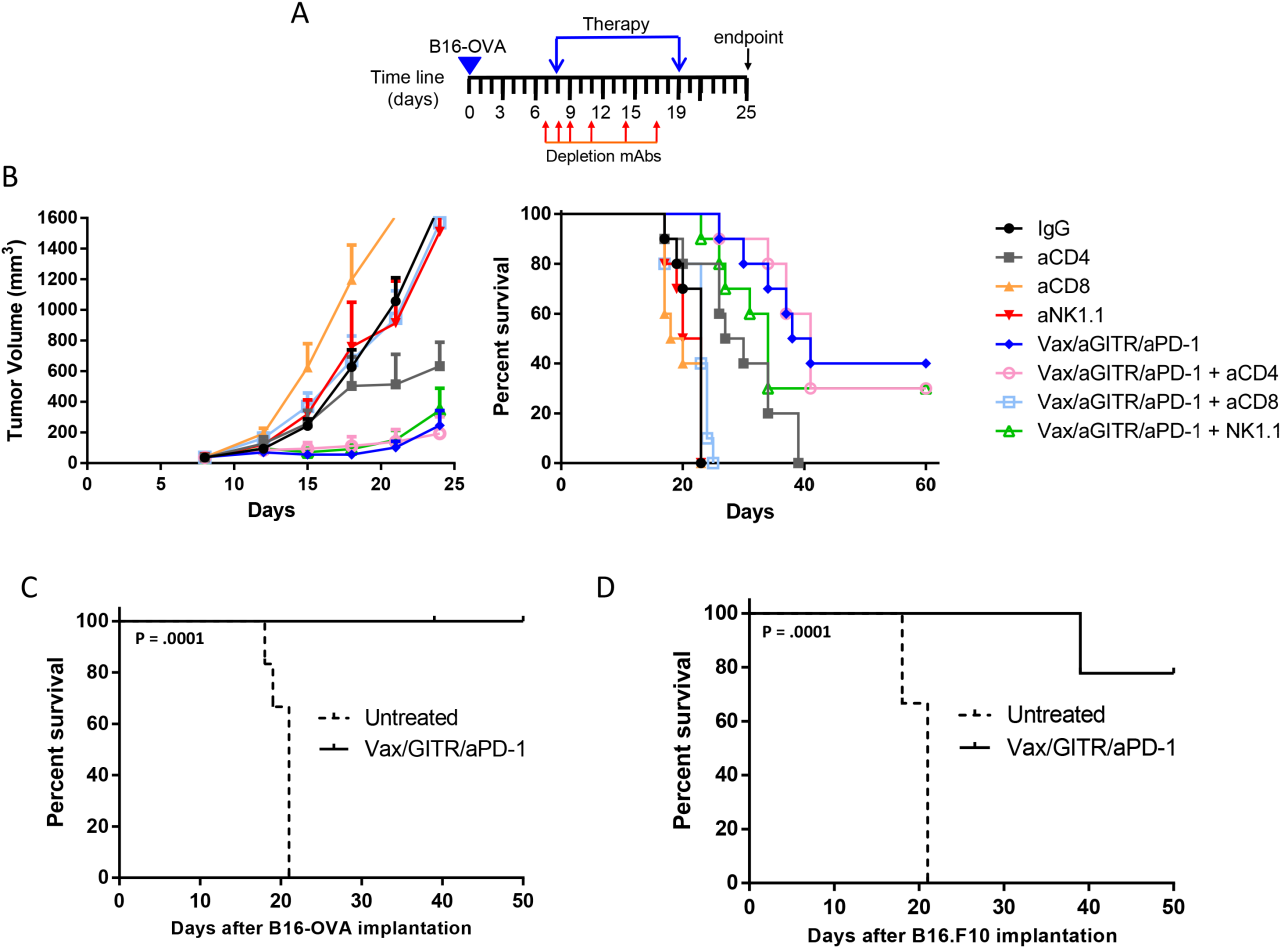
Vax/aGITR/aPD-1 efficacy depends on CD8^+^ T cells and treatment induces long-term memory **(A)** Dosing schedule for the therapeutic depletion study. B6 mice (n = 10/group) were injected s.c. with 4×10^5^ B16-OVA tumor cells and when tumor diameters reached ~40 mm^3^ they were depleted of CD8 cells, CD4 cells, or NK cells by administration of 200 μg mAb per mouse at days 7, 8, 9, 11, 14, 17; day 8 is the day when treatment with Vax/aGITR/aPD-1 or IgG started. Vaccine was dosed on day 8; aGITR on day 8 and 14; aPD-1 on day 10, 13, 16, and 19 post-tumor implantation. **(B)** Tumor volume and survival were monitored twice a week (mean +/− SEM). **(C-D)** Tumor-free mice (n = 6-9 per group) after combination treatments were re-challenged with B16-OVA (2×10^5^; **(C)** or B16.F10 (1.5×10^5^; **(D)**) cells on the same flank six months after primary tumor rejection. Age-matched mice were used for re-challenge controls. Results are representative of 2-3 independent experiments.

Tumor-infiltrating CD8^+^ T cells showed a synergistic enhancement against an immunizing peptide in the Vax/aGITR/aPD-1 combination therapy, indicating that the superior induction of potent CTL responses was most likely critical for the efficacy of the combination therapy. Therefore, we investigated the relevance of the effector populations on tumor rejection induced by the combination therapy. In a therapeutic setting, CD8^+^ T cells, CD4^+^ T cells, and NK cells were depleted in tumor-bearing mice as illustrated in Figure 5A. Our results showed that CD8 depletion completely abrogated the beneficial effects provided by Vax/aGITR/aPD-1, as no mice survived past 22 days post-implantation (Figure 5B). In contrast, the depletion of CD4 and NK cells did not inhibit the antitumor activity of Vax/aGITR/aPD-1 therapy (Figure 5B) by day 25 post tumor implantation, indicating these cells played minor roles in the efficacy observed. Overall, there was no statistical difference in tumors from control mice or those treated with aCD8 alone or aNK1.1 alone. In accordance with a previous study [34], we observed a delay in tumor growth and a significant difference in the observed survival (p=0.0037; CD4-depleted vs. Isotype) with the group treated with aCD4 alone (Figure 5B). However, there was no added benefit of administering aCD4 (Figure 5B) or aCD25 (Supplementary Figure 2) with the combination Vax/aGITR/aPD-1 therapy, suggesting that the combination can act independently of helper T cells or depletion of regulatory CD4^+^ T cells. Overall, the results demonstrate that CD8^+^ T cells are the main effector population responsible for eliciting tumor rejection.

The ultimate goal of both vaccination and active immunotherapy against cancer is the generation of long-lasting memory T cells, which can rapidly respond to subsequent Ag exposure. To assess memory responses, re-challenge experiments were carried out in tumor-free surviving animals, 6 months after completing treatment. All the mice that survived the first tumor challenge with Vax/aGITR/aPD-1 treatment survived a second tumor challenge against the same tumor 6 months later (Figure 5C), indicating durable antitumor immunity and induction of long-term memory responses. More interestingly, when mice cured after treatment with Vax/aGITR/aPD-1 were rechallenged with the parental B16.F10 tumor strain, which does not express OVA, ~80% of the mice remained tumor free, rejecting the tumor on re-challenge (Figure 5D). Overall, these data suggest that the combination Vax/aGITR/aPD-1 therapy can induce long-term memory responses, as well as epitope spreading against other antigens expressed by tumor cells.

### Combination Vax/aGITR/aPD-1 elicits potent Ag-specific tumor infiltrating KLRG1^+^ effector CD8^+^ T cells

**Figure 6:**
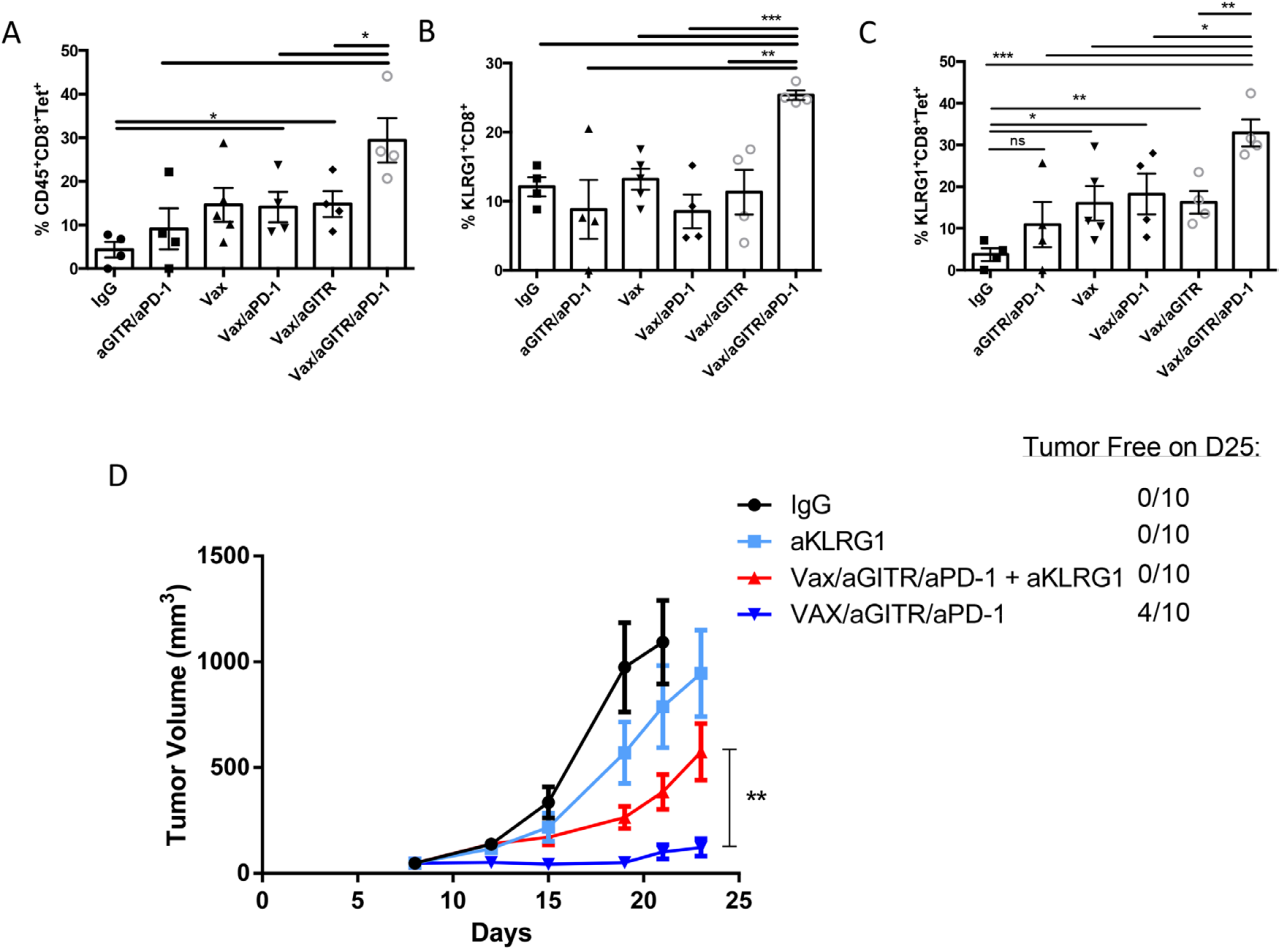
Combination Vax/aGITR/aPD-1 therapy expands tumor-specific CD8^+^ TILs and induces tumor clearance mediated in part by KLRG1^+^ effector-memory CD8^+^ T cells **(A)** representative scatter plot graphs show the percentage of H2-K^b^-SIINFEKL-restricted OVA-specific CD8^+^ T-cells, **(B)** percentage of KLRG1^+^CD8^+^ TILs (derived from CD45^+^ cells), and **(C)** the percentage of tetramer-binding KLRG1^+^CD8^+^ TILs 15 days after tumor inoculation (4-5 mice/group). **(D)** B6 mice (10 per group) were injected s.c. with 4×10^5^ B16-OVA tumor cells and at day 8 when tumor diameters reached ~50 mm^3^, therapy was initiated as in Figure 5A. 200 μg of aKLRG1 mAb was administered on days 7, 8, 9, 11, 14, 17, 20; day 8 was when therapeutic treatment started. Tumor volume and survival were monitored twice a week. Overall, graphs depict the mean+/− SEM of at least two independent experiments. All cell counts are relative and not absolute. *P<0.05; **P<0.01; ***P<0.001.

Extensive research in the field has demonstrated that CTLs play a major role in tumor rejection, and the numbers of tumor-infiltrating effector CD8+ T cells are often correlated with a good prognosis [35–37]. More recently, several studies have begun to support the hypothesis that the subset of KLRG1^+^ effector memory CD8^+^ T cells may predict therapeutic efficacy against pathogens and tumors [26–32, 38]. The increase of KLRG1^+^CD8^+^ T cells in the peripheral blood of non-tumor bearing mice in Figure 1F suggested these cells may be an immune correlate for the complete tumor regression elicited by the triple combination therapy (Figure 2). Thus, we examined if tumor regression was associated with the triple therapy's ability to drive robust tumor infiltrating KLRG1^+^ effector memory Ag-specific CD8^+^ T cell responses. Twelve days after tumor implantation (5 days after the start of therapy) (Figure 2A), we first noted that the combination Vax/aGITR/aPD-1 therapy had the highest increase of tetramer-specific CD8^+^ T cell responses in the tumors (Figure 6A). Then, we evaluated the effector memory CD8^+^ T cell subset based on the expression marker KLRG1. Interestingly, the Vax/aGITR/aPD-1 therapy resulted in a ~2-fold increase in the frequency of tumor-infiltrating KLRG1^+^CD8^+^ effector cells and KLRG1^+^CD8^+^Tet^+^ cells, compared to all other groups (Figure 6B-6C), inferring that Ag-specific KLRG1^+^CD8^+^ effector cells can traffic to the tumor site to elicit rapid effector function. Overall, we demonstrated that generating higher KLRG1^+^CD8^+^ effector T cells correlated with the remarkable regression of established tumors seen in the combination Vax/aGITR/aPD-1 therapy.

If the expansion of the KLRG1^+^CD8^+^ subset population is an additional potential mechanism that helped establish better tumor growth control/regression in the combination Vax/aGITR/aPD-1 therapy, we wanted to determine whether targeting the KLRG1^+^CD8^+^ effector T cell subpopulation would lead to a loss of tumor growth control. Prior to performing a therapeutic efficacy study, we determined if the anti-KLRG1 (aKLRG1) antibody could reduce the target population. To examine this, two groups of non-tumor bearing mice were vaccinated with the combination Vax/aGITR/aPD-1 therapy and one group was treated with 200 μg of aKLRG1 mAb (200 μg) at day 0, 2, 4, and 6 post-vaccination, and at day 7 after therapy initiation the expression of KLRG1 was monitored on CD8^+^ T cells from the blood and spleen (Supplementary Figure 3). We observed that the aKLRG1 mAb reduced the percentage of CD8^+^ T cells (Supplementary Figure 3A) and depleted the target KLRG1^+^CD8^+^ population (Supplementary Figure 3B-3C). The Vax/aGITR/aPD-1 treated aKLRG1 mice resulted in a significant decrease in the frequency and/or absolute total number of KLRG1^+^CD8^+^CD44^+^ and KLRG1^+^CD8^+^Tet^+^ populations in the blood and spleen, compared with the non-treated aKLRG1 control group (Supplementary Figure 3B). Next we assessed the contribution of the KLRG1^+^CD8^+^ population at facilitating tumor rejection induced by the triple combination therapy by depleting KLRG1^+^CD8^+^ cells in tumor-bearing mice. Our results revealed that targeting KLRG1 significantly reduced protection as mice depleted with KLRG1 mAb showed faster tumor growth than the combination treated without KLRG1 mAb (Figure 6D). More strikingly, the combination therapy with aKLRG1 mAb no longer established tumor regression and long-term survival over combination therapy without aKLRG1 treatment (0% vs 40% tumor rejection). Taken together, these results suggest that the increase of Ag-specific KLRG1^+^ effector CD8^+^ T cells induced by the triple combination was a mechanism by which it facilitated tumor growth control, regression, and long-term survival in this melanoma therapeutic model. Thus, the expansion of such an effector CD8^+^ T cell subpopulation could be a major benefit for future cancer immunotherapeutic strategies.

## DISCUSSION

Immune checkpoint blockade antibodies have shown promising clinical benefit in cancer patients, highlighting a major breakthrough in the fight against cancer. However, monotherapies have limited efficacy in improving outcomes and benefit only a subset of patients. It has been proposed that such immunotherapies are unable to overcome T cell anergy because they do not specifically target and expand tumor-reactive T cells [19]. Thus, one approach to overcome this limitation would require the administration of these therapies with an Ag-specific vaccine. Vaccines can drive effective CD8 T cell responses and long-term memory in tumor models, making them a promising therapeutic strategy to combat cancer. Therefore, we hypothesized that a combination aGITR/aPD-1 therapy with vaccination would induce the expansion of tumor-reactive CD8 T cells and thus elicit sufficient tumor control and regression in a poorly immunogenic tumor model. Here, we demonstrated that a single vaccine immunization with combination aGITR/aPD-1 therapy substantially enhanced Ag-specific polyfunctional CTL responses in the tumor, with a concomitant reduction in the frequency of Tregs in the tumor. This resulted in 50% tumor rejection in established melanoma tumor-bearing mice. Finally, we showed that the therapeutic efficacy was associated with the increase in the magnitude and phenotype of potent tetramer-specific, effector memory CD8^+^ T cells.

GITR is expressed at low levels on resting CD4^+^ and CD8^+^ T cells and up-regulated following T cell activation [12–13]. Ligation of GITR is known to provide a costimulatory signal that enhances T cell proliferation and effector functions [11–13]. Furthermore, GITR expression on CD8^+^ T cells is required to boost CD8^+^ T cell expansion and help sustain their survival following therapy in a vaccine setting [11–13]. On the other hand, the PD-1 pathway is known to mediate T cell exhaustion; blocking this pathway has proven to be sufficient to reinvigorate both murine and human T cells. By targeting GITR and PD-1 during vaccination, effector T cells can be amplified and their function sustained/reinvigorated within the tumor. Together these mechanisms explain the enhanced number of Th1 cytokine-producing CD8^+^ T cells in the tumor and spleen, as well as the robust increase of Ag-specific tumor-infiltrating effector CD8^+^ T cell responses with cytolytic potential (Figures 1, 3 & 6). The increase in the number of Th1 cytokine-producing CD8^+^ T cells, shifting a suppressive TME to a more inflammatory state, likely contributed to a more effective antitumor response [39–40]. The induction of cytolytic CD8^+^ T cells is considered to be essential for controlling and eliminating established tumors [30–32]. Thus, the administration of the Vax/aGITR/aPD-1 therapy in our study led to markedly better inhibition of tumor growth, tumor clearance, and prolonged survival in 50% of the treated mice. Moreover, depletion of CD8^+^ T cells in mice nullified this antitumor activity produced by the combination Vax/aGITR/aPD-1 therapy, supporting the conclusion that the antitumor activity was dependent on CD8^+^ T cells. Specifically, antitumor activity was associated with elevation of potent tumor-specific T cells in the B16-OVA tumor model.

In addition to aGITR's positive effects on effector CD8^+^ T cells, recent evidence shows that using GITR-targeted antibodies can abrogate the suppressive effect of Tregs in the tumor [11–13, 41]. This aspect of GITR is valuable to target, as limiting the Treg population *in vivo* promotes better-primed immune responses and antitumor immunity. Here, we demonstrated that combination Vax/aGITR/aPD-1 therapy reduced intratumoral Treg frequency, providing further explanation for the improved tumor efficacy observed with the combination Vax/aGITR/aPD-1 treatment. This conclusion is supported by the increase in the CD8/Treg ratio in the combination Vax/aGITR/aPD-1 therapy (Figure 4C). Increased CD8/Treg ratios have been associated with sensitizing tumors to a given therapy and improved survival in patients with cancers [33, 42–43]. The notable increase in the CD8^+^ T cell to Treg ratio within the tumor correlated with better tumor suppression and promoting inflammation in the TME for mediating tumor rejection. Although aGITR administered as a monotherapy or as a dual therapy reduced Tregs (Figure 4), we did not observe improved tumor suppression or synergy when CD4 depletion was combined with Vax/aGITR/aPD-1 therapy. Therefore, a contribution from CD4^+^ T cells cannot be ruled out because overall long term survival was slightly decreased (Figure 5B). The precise anit-GITR mechanism of action are controversial as GITR targeting are thought to either only effect Tregs or act directly on CD8 T cells [11–17]. It is likely that GITR has dual roles, both Treg tumor depletion and CD8 T cell costimulatuory signaling [44]. However, this is an area of ongoing study we are currently investigating. Moreover, it has been demonstrated that the interaction of GITR expression by responder T cells with its ligand (GITRL) on APCs can enhance T cell survival, expansion, and differentiation into effector cells [45]. Collectively, these results suggest that therapeutically targeting or manipulating the GITR-GITRL pathway provides strong rationale for unique approaches to cancer immunotherapy and for their potential combination with other TNFR agonists (e.g. CD137, OX40) [46–47].

While depletion of Tregs is useful to inhibit tumor growth as described above, this aspect alone is not sufficient to induce complete tumor regression. In our study, we saw that aGITR/aPD-1 therapy without the vaccine did increase antitumor immunity by significantly reducing the frequency of Tregs in the tumor similar to the Vax/aGITR/aPD-1 therapy (Figure 4); however, the efficacy of aGITR/aPD-1 was relatively weak compared to the combination Vax/aGITR/aPD-1 therapy (Figure 2C). We find these results in agreement with Lu *et al*., reporting that aGITR/aPD-1 combination can synergize to enhance immunity, but is not enough to drive complete tumor clearance [18]. And, to better enhance optimal antitumor effects in aggressive tumor models it requires synergy with an additional immunotherapy. Although Lu *et al*. similarly demonstrated that aGITR/aPD-1 combination can enhance CD8^+^ T cells and reduce Tregs, we further revealed that aGITR/aPD-1 combination can enhance Ag-specific plurifunctional effector CD8^+^ T cells responses when combined with a vaccine. The difference between the two groups (aGITR/aPD-1 vs Vax/aGITR/aPD-1) is best attributed to the lack of induced tumor-reactive T cells in the TME in the aGITR/aPD-1 combination, as it was not able to induce potent Ag-specific CTLs compared to the combination Vax/aGITR/aPD-1 therapy (Figures 3 and 6B). This underscored the necessity of combining PD-1 blockade and GITR triggering with a vaccine to elicit a potent optimal antitumor effect. We find this in accordance with previous studies, demonstrating that mAb therapies delivered in the absence of specific antigen in poorly immunogenic tumor models are ineffective at expanding target specific tumor-reactive T cells [19]. It is only when an immunotherapy can prime and drive potent Ag-specific CTL responses that it is capable of mediating tumor clearance, thus leading to better therapeutic efficacy (Figure 2). Nevertheless, given that the triple combination therapy only led to 50% cure rate suggest there are additional immunosuppressive mechanisms (e.g. other checkpoints) at play that are preventing cures in all the mice. This is an area of further investigation.

While each component of the combination therapy plays an essential role individually, the T cell potential therapies (Vax, Vax/aPD-1, and Vax/aGITR) provided no more than 20% tumor clearance (Figure 2). These results suggest that a single or double combination alone may not be sufficient to overcome the multiple resistance mechanisms elicited by the TME of more aggressive or non-immunogenic tumors. Data has demonstrated that peptide vaccines can add little additional benefit when combined with checkpoint inhibitors [9, 48–49]. However, as suggested, the limited benefit of adding peptide vaccines may not be due the selected tumor-associated antigen target, because differentiation antigens are highly expressed in most melanoma tumors [9, 50]. Thus, determining the right formulation of combination immunotherapies and/or adjuvants will be crucial to maximize patient outcome [51–52]. Our data support using more than two immunomodulatory therapeutic strategies to overcome different tumor immunosuppressive pathways. Collectively, these results demonstrate that triple Vax/aGITR/aPD-1 combination therapy harnesses the therapeutic potential to enhance cytolytic CD8^+^ T cells, while at the same time reducing Tregs. Additional studies are warranted to further define the synergy mechanisms in the triple combination [17].

The goal of cancer immunotherapies is the induction of the most potent subsets of memory CD8 T cell populations to rapidly control or clear tumors. Here we demonstrated that our combination Vax/aGITR/aPD-1 therapy induced both Ag-specific EM and CM CD8^+^ T cells, and uniquely amplified an effector KLRG1 phenotype memory. Several studies have begun to show that effector-memory KLRG1^+^CD8^+^ T cells might be essential for rapid regression of established subcutaneous tumors [29–32]. Here, we show that the enhanced induction of tumor-specific KLRG1^+^CD8^+^ effector memory T cells in the blood, spleen, and tumors correlated with the better efficacy of the Vax/aGITR/aPD-1 treated groups against established melanoma tumors (Figures 1F and 6). Moreover, a key finding from our study demonstrated that the increase of tumor-infiltrating Ag-specific CD8^+^ T cells with KLRG1^+^ effector phenotype can play a role in eliciting tumor clearance in the combination Vax/aGITR/aPD-1 therapy. Reduction of KLRG1^+^CD8^+^ T cells in tumor-bearing mice significantly attenuated the tumor clearance effects of Vax/aGITR/aPD-1 therapy and allowed the tumors to grow larger compared to the non-treated KLRG1 mAb therapeutic group (Figure 6D). Natural killer (NK) cells are known to express KLRG1, however, it is unlikely that in this model NK cells played a central role in tumor efficacy (Figure 4). Therefore, we showed here for the first time that the degree and quality of melanoma-associated effector memory KLRG1^+^CD8^+^ T cells can play an important role for controlling and/or resolving tumors. These results are consistent with the observations that effector memory T cells can migrate quickly to the tumor-site and initiate rapid effector function [53]. Collectively, we find these findings in accordance with previous data highlighting that a predominant KLRG1^+^ effector-memory T cell response can be a vital correlate of immunity for the efficacy of therapeutic cancer vaccines or other immunotherapies [29–31]. However, the manner in which combination Vax/aGITR/aPD-1 therapy is able to preferentially skew and expand the frequency of KLRG1^+^ effector memory T cell responses is not yet entirely clear [54–56]. Further studies are needed to elucidate these mechanisms and ongoing studies in our laboratory may provide answers to these important questions. Overall, the identification of how to modulate the expansion of this population and/or other potent non-KLRG1 CD8^+^ T cell subsets may prove beneficial for the development of future effective cancer immunotherapies.

The generation of long-lasting memory CD8^+^ T cells is the ultimate goal of active immunotherapies against cancer, as it has the potential to provide protection from tumor growth over time. Here, we showed mice that rejected tumors after treatment remained protected against a challenge from the same tumor, indicating the establishment of long-lasting memory elicited by the triple combination therapy. This notion is supported by the ability of the Vax/aGITR/aPD-1 combination therapy to enhance central memory CD44^+^CD62L^+^ CD8^+^ T cells during vaccination (Figure 1E) [57–59]. It demonstrates that the triple combination therapy can induce CM responses and that their establishment is not negatively affected. In addition, it has also been demonstrated that a subpopulation of CD8^+^ T cells expressing CD127^+^ and KLRG1^−^ can also be long-lived memory cells [60]. Therefore, one might also expect that the recall responses in Figure 5, in mice that had cleared tumors would require the establishment of CD127^+^KLRG1^−^ CD8^+^ T cells. We are currently investigating the ability of Vax/aGITR/aPD-1 therapy to induce different degrees of heterogeneity of central memory CD8^+^ T cells. Additionally, our therapy showed it could induce epitope spreading, as ~80% of the cured mice remained protected even when rechallenged against parental B16F10 that lacks the antigen used in the therapeutic vaccine. This strategy could therefore be useful to augment antitumor immunity against both self- and non-self tumor antigens. Overall, our findings provide a scientific basis for the combination of vaccines with dual aGITR/aPD-1 therapy in future clinical trials.

## MATERIALS AND METHODS

### Animals and tumor cells

Female, 6 to 8 weeks old C57BL/6 (B6) mice were purchased from Jackson Laboratories (Bar Harbor, ME). All mouse procedures were performed in accordance with protocols approved by the Janssen Pharmaceuticals IACUC (Spring House, PA). The B16-F10 (CRL-6475) mouse melanoma cell line was purchased from ATCC (Manassas, VA). The B16F10-OVA (B16-OVA) cell line was obtained from K. Rock (University of Massachusetts Medical School). The B16-F10 and B16-OVA cell lines were maintained as detailed in the Supplementary Material and Methods.

### Reagents

Peptides OVA_257-264_ and OVA_323-339_ were purchased from MBL International and GenScript. Poly (I:C) and CpG were obtained from Invivogen and reconstituted according to manufacturer's protocol. Anti-mouse GITR antibody (aGITR, clone DTA-1), anti-mouse CD279 antibody (aPD-1, clone RMP1-14), anti-mouse CD4 (aCD4, clone GK1.5), anti-mouse CD8 (aCD8, clone 53-6.72), anti-mouse CD25 antibody (aCD25, clone PC-61.5.3), anti-mouse NK1.1 (aNK1.1, clone PK136), anti-mouse/human killer cell lectin-like receptor subfamily G, member 1 (aKLRG1, clone 2F1; hamster antibody) and control antibodies (rat IgG2A, Clone 2A3; rat IgG2b, Clone LTF-2; rat IgG1, clone HRPN) and hamster IgG (BE0087) were purchased from BioXcell (West Lebanon, NH).

### Tumor models, tumor vaccine, and treatment

B16-OVA (400,000 for challenge and 200,000 for rechallenge) and B16-F10 (150,000) tumor cells were implanted subcutaneously (s.c.) in the right flank of mice. Tumor vaccine consisted of adjuvants Poly (I:C) (100 μg/mouse), and CpG (ODN1826, 5 mM/mouse) plus OVA CD4-helper (ISQAVHAAHAEINEAGR) peptide (323-339; 20 μg/mouse) and OVA CD8-restricted (SIINFEKL) peptide (257-264; 20 μg/mouse). Adjuvants Poly (I:C) and CpG activate DCs and help induce cross-presentation [61–62], and therefore were selected to facilitate better T cell priming. Mice were immunized with 200 μl of vaccine mixture s.c. on indicated days. For therapeutic treatment, mice were treated with intraperitoneal (i.p.) injections of PD-1 blocking antibody (200 μg/mouse/injection), GITR targeting antibody (200 μg/mouse/injection), and control IgGs (rat IgG2b and rat IgG2a) along with the peptide vaccine that were dosed as described in Figures 2a, 5a. Administration strategy of aPD-1 and aGITR were adapted from the following references [18, 63]. For *in vivo* cell depletion, anti-CD4 mAb (0.2 mg/dose), anti-CD8 mAb (0.2 mg/dose), anti-NK1.1 mAb (0.2 mg/dose), and anti-KLRG1 mAb (0.2 mg/dose) were injected i.p. following the schedules shown in Figure 5A. Animals were monitored for tumor growth twice a week using electronic calipers. Tumor volumes were calculated according to the formula: V = (length x width^2^)/2. For survival experiments, mice were euthanized when tumor size reached 2000 mm^3^.

### Flow cytometry

Lymphocytes were isolated and processed from the spleen, peripheral blood, and tumors as previously described [31]. The antibodies used in the present study are listed in Supplementary Materials and Methods.

### TIL Isolation

Tumor-infiltrating lymphocytes (TILs) were harvested and collected on day 12 or day 15 by dissection of tumors into small fragments followed by digestion in 1 mg/ml collagenase IV and 5 mg/ml DNase (Sigma) in PBS for 30 minutes at 37°C as described [64]. After filtration through nylon mesh, lymphocytes were stained and analyzed by flow cytometry as described [31].

### Statistical analysis

Statistical significance was determined by unpaired Student t test (two-tailed) and Kaplan-Meier survival where appropriate, to analyze the cellular immune responses and tumor measures. Error bars indicate standard error of the mean (SEM) and all graphs and statistical analysis were generated using Prism 6 software (GraphPad Software, Inc.). ***, P<0.001; **, P<0.01; *, P<0.05.

## SUPPLEMENTARY MATERIALS FIGURES


