# Supplementary material for: Combination GITR targeting/PD-1 blockade with vaccination drives robust antigen-specific antitumor immunity
Source: Oncotarget. 2017 Mar 27;8(24):39117–30. doi: 10.18632/oncotarget.16605 (PMC5503599; doi:10.18632/oncotarget.16605)
Supplement: Supplementary file 1 [file oncotarget-08-39117-s001.pdf]

Combination GITR targeting/PD-1 blockade with vaccination drives robust antigen-specific antitumor immunity

SUPPLEMENTARY MATERIALS

Tumor cell culturing

B16-F10 cells were maintained in DMEM supplemented with 10% FBS in a humidified atmosphere with 5% CO_2 at 37°C. The B16-OVA cell lines were maintained in Hybridoma culture (HCM) media, containing the following components: 10% FCS, 1% Penicillin/streptomycin, 1% 1-glutamine, 1% NEAA (non-essential amino acids), 1% Hepes and 1M 2-Merceptoethanol. All cell lines were determined to be *Mycoplasma* free and authenticated.

ELISPOT assays

Spleens were harvested 7 days following immunization and cells producing IFNγ from immunized mice were enumerated by ELISpot assay as described previously [1]. For T cell responses, splenocytes were stimulated with the peptide OVA (257-264; 2.5 µg/mL). The ELISpots were counted using Autoimmun Diagnostika GMBH (AID) ELISpot Reader.

Flow cytometry

Lymphocyes were isolated and processed from the spleen, peripheral blood and tumors as previously described [1]. Cell surface molecule staining, MHC class I peptide tetramer staining, intracellular staining, and flow cytometry were done as previously described [1]. Briefly, cells were incubated for 30 min at 4°C with CD45, CD4, CD8, CD44, CD62L, KLRG1 (clones: 2F1, 2F1/KLRG1, 14C2A07), CD25, LIVE/Dead fixable violet dead cell stain kit, and MHC class I peptide tetramer to H2-K^b-SIINFEKL-OVA. All antibodies were obtained from eBioscience, BD Biosciences, Biolegend, and MBL International. Intracellular cytokine staining was perfomed after 5 hours of *ex vivo* stimulation with 2.5 µg/ml of $OVA_{257-264}$ $CD8^+$ peptide (SIINFEKL) in the presence of GolgiStop and GolgiPlug (Ebioscience) with or without the CD107a FITC antibody (degranulation marker) for 5 hours. For intracellular staining, cells were fixed and permeabilized with either the Biolegend or ebioscience FoxP3 staining buffer kit according to the manufacturer's instructions. Cells were incubated for 45 min at 4°C with antibodies to IL-2, TNFα, IFNγ, CD3, and FoxP3. Cells were collected and analyzed using the Fortessa flow cytometer using DIVA (BD Biosciences) and analyzed using FlowJO software (Tree Star, Ashland, OR) and SPICE v5.3 (free available from http://exon.niaid.nih.gov/spice/).

REFERENCES

1. Villarreal DO, Wise MC, Siefert RJ, Yan J, Wood LM, Weiner DB. Ubiquitin-like molecule ISG15 acts as an immune adjuvant to enhance antigen-specific CD8 T cell tumor immunity. Molecular Therapy 2015; 10:1653-1662.

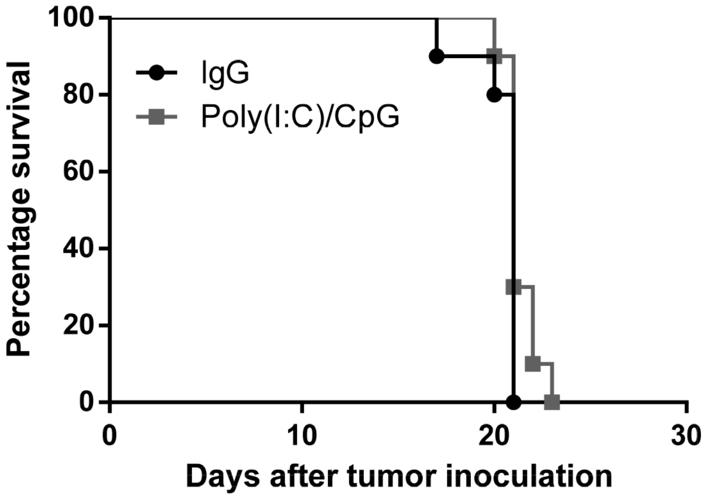

Supplementary Figure 1: Adjuvants Poly(I:C)/CpG do not suppress tumor growth in B16-OVA tumor model. B16-OVA established tumors (~30-40 mm^3) were treated s.c. on day 7 with combination Poly(I:C)/CpG. Survival was monitored as shown. Results are representative of two independent experiments with 10 mice per group.

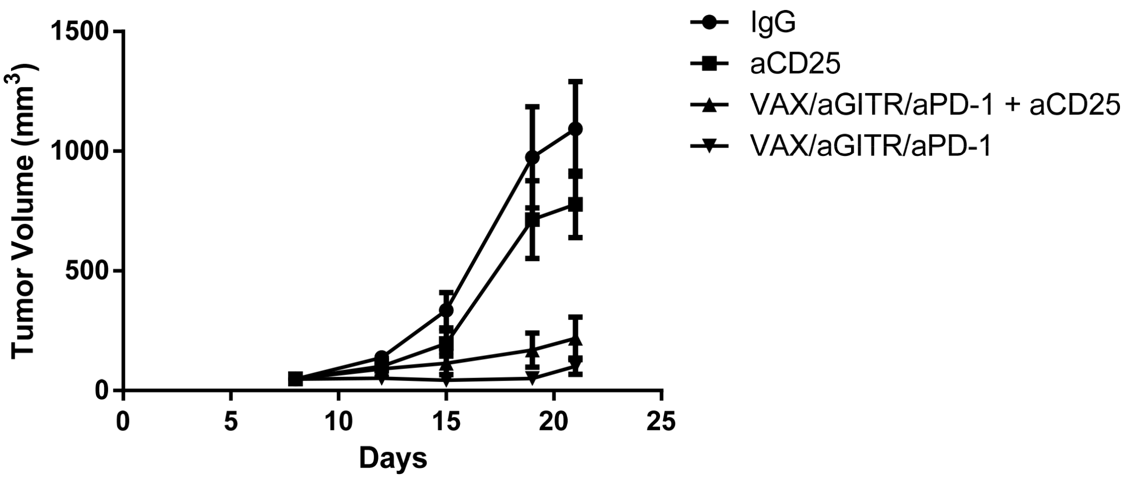

Supplementary Figure 2: Depletion of CD25^{+} cells with combination Vax/aGITR/aPD-1 therapy does not abrogate or enhance tumor efficacy. Combination treatment and dosing of 200 μg of anti-CD25 were delivered as illustrated in Figure 5A. Tumor volume was monitored twice a week (mean +/- SEM are plotted). Results are representative of two independent experiments with 10 mice per group.

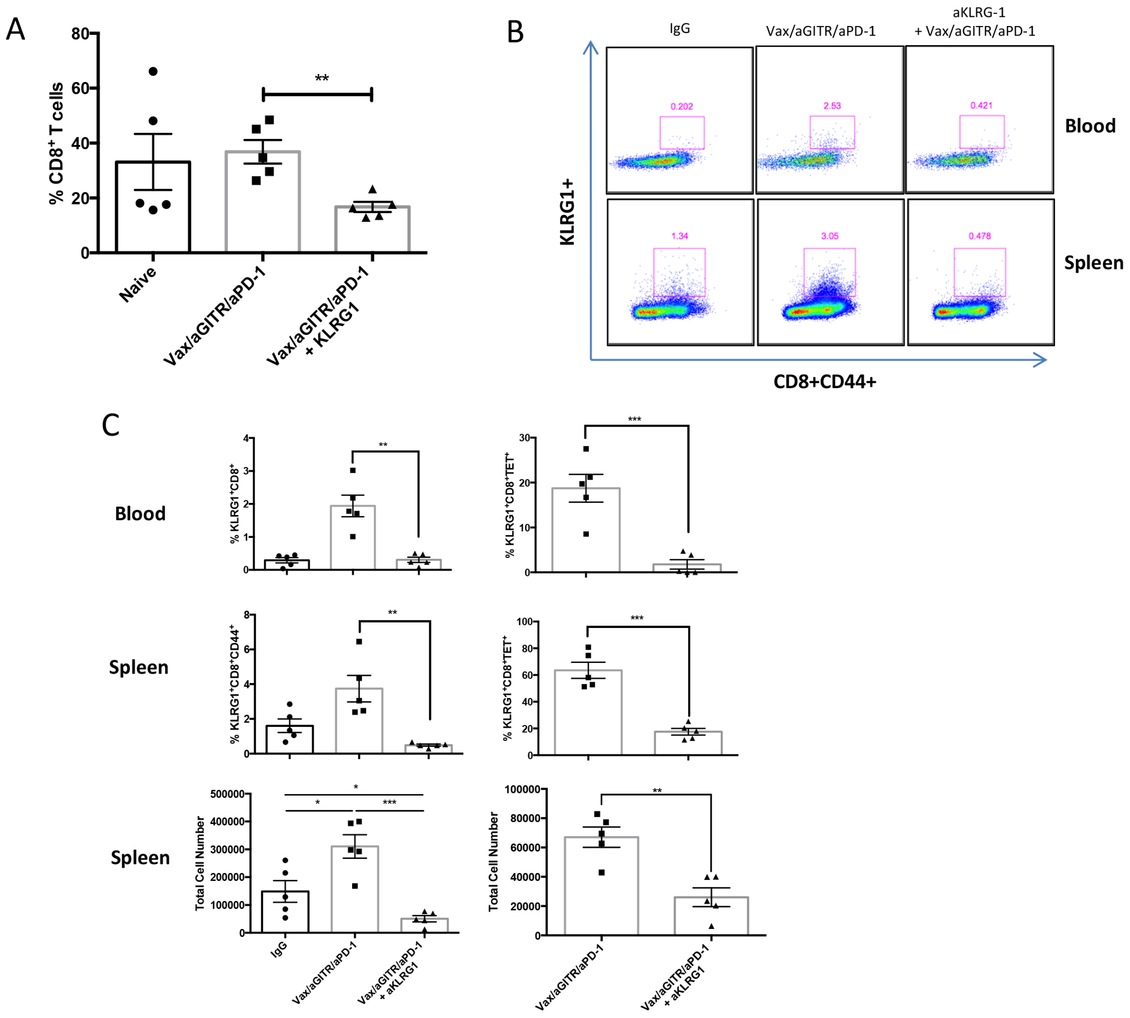

Supplementary Figure 3: Anti-KLRG1 targeted monoclonal antibody reduces KLRG1^{+}CD8^{+} target population. (A-B) Naïve tumor-free mice were dosed with Vax/aGITR/aPD-1 combination and isotype as in Figure 1. The anti-KLRG1-treated mice were administered 100 µg of anti-KLRG1 mAb on 2, 4, and 6 days post vaccination. Mice were sacrificed on day 7 post vaccination and lymphocytes from both the blood and spleens were collected to assess expression ofCD8, KLRG1, and CD44. **(A)** the percentage of CD8^{+} T cells in the spleen after treatment with anti-KLRG1 antibody. **(B)** Representative flow plots showing percentages of KLRG1^{+}CD8^{+} in the blood and spleen and **(C)** compiled data of the frequency and/or total cell numbers of KLRG1^{+}CD8^{+}CD44^{+} and KLRG1^{+}CD8^{+}Tet^{+} cells (left and right panels, respectively) in the blood and spleen. Results are representative of 2 independent experiments with 5 mice per group. *P<0.05; **P<0.01; ***P<0.001. Error bars indicate SEM.
